# *Caenorhabditis elegans* POT-1 and POT-2 Repress Telomere Maintenance Pathways

**DOI:** 10.1534/g3.112.004440

**Published:** 2013-02-01

**Authors:** Ludmila Shtessel, Mia Rochelle Lowden, Chen Cheng, Matt Simon, Kyle Wang, Shawn Ahmed

**Affiliations:** * Department of Genetics, University of North Carolina, Chapel Hill, North Carolina 27599-3280; † Curriculum in Genetics and Molecular Biology, University of North Carolina, Chapel Hill, North Carolina 27599-3280; ‡ Department of Biology, University of North Carolina, Chapel Hill, North Carolina 27599-3280

## Abstract

Telomeres are composed of simple tandem DNA repeats that protect the ends of linear chromosomes from replicative erosion or inappropriate DNA damage response mechanisms. The mammalian Protection Of Telomeres (POT1) protein interacts with single-stranded telomeric DNA and can exert positive and negative effects on telomere length. Of four distinct POT1 homologs in the roundworm *Caenorhabditis elegans*, deficiency for POT-1 or POT-2 resulted in progressive telomere elongation that occurred because both proteins negatively regulate telomerase. We created a POT-1::mCherry fusion protein that forms discrete foci at *C. elegans* telomeres, independent of POT-2, allowing for live analysis of telomere dynamics. Transgenic *pot-1*::*mCherry* repressed telomerase in *pot-1* mutants. Animals deficient for *pot-1*, but not *pot-2*, displayed mildly enhanced telomere erosion rates in the absence of the telomerase reverse transcriptase, *trt-1*. However, *trt-1*; *pot-1* double mutants exhibited delayed senescence in comparison to *trt-1* animals, and senescence was further delayed in *trt-1*; *pot-2*; *pot-1* triple mutants, some of which survived robustly in the absence of telomerase. Our results indicate that POT-1 and POT-2 play independent roles in suppressing a telomerase-independent telomere maintenance pathway but may function together to repress telomerase.

Human somatic cells have finite replicative lifespans and can enter an irreversible cell-cycle arrest, termed senescence, in response to various stresses. Senescence can occur due to progressive shortening of telomeres, which cannot be completely replicated by canonical DNA polymerases (Harley *et al.* 1990). Telomeres are composed of simple TTAGGG repeats in vertebrates and related sequences in other organisms, such as TTAGGC repeats in *Caenorhabditis elegans*. To combat telomere erosion, cells can express the enzyme telomerase, which adds *de novo* telomere repeats to chromosome ends via reverse transcription from an RNA template (Greider and Blackburn 1989). Telomerase is expressed at high levels in germ cells and can be expressed in human somatic cells, but its expression is transient or absent altogether in more differentiated cell types (Kim *et al.* 1994; Sharma *et al.* 1995).

The shelterin complex, composed of six mammalian telomere-binding proteins TRF1, TRF2, TIN2, POT1, RAP1, and TPP1, and its associated proteins protect telomeres from nucleases and DNA damage repair mechanisms that can lead to exacerbated telomere shortening or cellular senescence (Diotti and Loayza 2011). Shelterin components maintain telomere homeostasis by positively and negatively regulating telomere length. The double-stranded telomeric DNA-binding proteins TRF1 and TRF2 have been implicated as negative regulators of telomere length, where removal of TRF1 from telomeres or overexpression of TRF2 yielded telomere elongation or erosion, respectively (Smogorzewska *et al.* 2000; van Steensel and de Lange 1997). TIN2 and TPP1 proteins bridge the interaction between these double-stranded telomere-binding proteins and the single-stranded telomere-binding protein, POT1, and are also considered negative regulators of telomere length as their depletion results in progressive telomere elongation (Kim *et al.* 1999; Ye and de Lange 2004; Ye *et al.* 2004).

Human Protection Of Telomeres 1 interacts with single-stranded telomeric DNA via two oligonucleotide/oligosaccharide (OB) folds and is primarily considered a negative regulator of telomere length (Kendellen *et al.* 2009; Veldman *et al.* 2004; Ye *et al.* 2004). However, in numerous studies researchers have revealed roles for POT1 in both telomere elongation and telomere protection. POT1 overexpression (Armbruster *et al.* 2004; Liu *et al.* 2004) and mutant or splice-variant POT1 expression (Armbruster *et al.* 2004; Colgin *et al.* 2003; Kendellen *et al.* 2009; Liu *et al.* 2004; Loayza and De Lange 2003) can elicit telomere elongation. In addition, POT1 can inhibit telomere repeat synthesis in the presence of its binding partner TPP1 but promotes telomerase processivity *in vitro* in its absence (Kelleher *et al.* 2005; Wang *et al.* 2007).

Both mouse Pot1 homologs promote chromosome end protection, as G-strand overhangs lengthen in Pot1b−/− cells, and end-to-end chromosome fusions occur as a result of telomere deprotection in both Pot1a−/− and Pot1b−/− cells (He *et al.* 2006, 2009; Hockemeyer *et al.* 2006, 2008; Wu *et al.* 2006). However, disparate cellular and telomere phenotypes have been reported. For example, fibroblasts derived from Pot1a−/− mice senesced prematurely in one study (Wu *et al.* 2006) but not in another (Hockemeyer *et al.* 2006). In addition, Pot1b−/− cells did not prematurely senescence in one study (Hockemeyer *et al.* 2006), but mouse embryonic fibroblasts overexpressing an OB-fold Pot1b mutant exhibited early-onset senescence in another study (He *et al.* 2006). Moreover, telomeres from Pot1b−/− cells have been shown to either shorten or stay the same (He *et al.* 2009; Hockemeyer *et al.* 2006, 2008), whereas Pot1a−/− cells exhibited telomere elongation (Wu *et al.* 2006).

The *C. elegans* genome is predicted to encode four proteins with OB folds homologous to mammalian POT1, including a single protein with an OB1 fold, POT-1, and three proteins with OB2 folds, POT-2, POT-3, and MRT-1 (Figure 1A) (Meier *et al.* 2009; Raices *et al.* 2008). Previous work has illustrated that POT-1, also known as CeOB2, and POT-2, also known as CeOB1, can interact with single-stranded telomeric DNA *in vitro* (Raices *et al.* 2008). In addition, this study reported elongated telomeres for both *pot-1(tm1620)* and *pot-2(tm1400)* mutant strains, although *pot-1(tm1620)* telomeres were distinctive and appeared similar to those of human cells that maintain their telomeres by a telomerase-independent telomere replication pathway termed alternative lengthening of telomeres (ALT).

**Figure 1:**
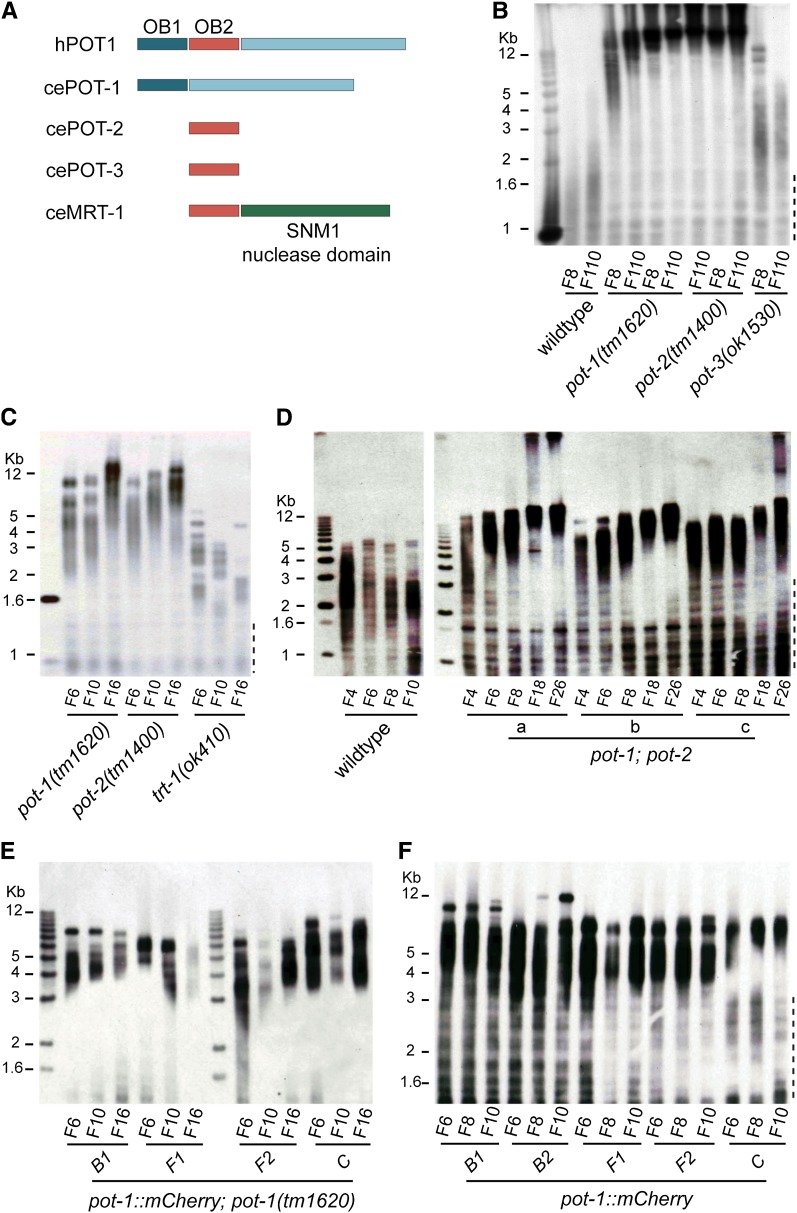
POT-1 and POT-2 are negative regulators of telomere replication. (A) A representation of the four POT-1 homologs in *C*. elegans. Terminal restriction fragment length analysis was performed on DNA collected from consecutive generations of (B) *pot-1(tm1620)*, *pot-2(1400)*, and *pot-3(ok1530)* single mutants, (C) outcrossed *pot-2(1400)* and *pot-1(tm1620)* single mutants, (D) wildtype and *pot-1*; *pot-2* double mutants, (E) outcrossed *pot-1*::*mCherry*; *pot-1(tm1620)* strains, and (F) outcrossed *pot-1*::*mCherry* strains. Dashed line to the right of the blots indicates internal telomeric sequences.

We previously demonstrated that one of four POT1 homologs, MRT-1, is necessary for telomerase-mediated telomere repeat addition *in vivo* (Meier *et al.* 2009). Here we investigate additional roles for these *C. elegans* proteins that contain POT1 OB folds by studying telomere dynamics in *pot-1* and *pot-2* mutants. We illustrate that both POT-1 and POT-2 are negative regulators of telomerase, indicating a similar and previously unknown role for these proteins in *C. elegans* telomere biology. We develop a *pot-1*::*mCherry* transgene that localizes to telomeres and represses telomerase *in vivo*. Additionally, we demonstrate a unique role for POT-1 in telomere protection and that POT-1 and POT-2 function non-redundantly to repress a telomerase-independent telomere maintenance pathway.

## Materials and Methods

### Strains

Unless noted otherwise, all strains were cultured at 20° on nematode growth medium plates seeded with *Escherichia coli* OP50. Strains used include Bristol N2 ancestral, CB61 *dpy-5(e61) I*, YA1059 *trt-1(ok410) I*, CB402 *unc-55(e402) I*, CB193 *unc-29(e193) I*, YA1197 *ypIn2 (Pdaz-1*::*pot-1*::*mCherry*::*tbb-2utr)*, YA1198 *ypIn3 (Pdaz-1*::*pot-1*::*mCherry*::*tbb-2utr)*, YA1024 *pot-2(tm1400) II*, CB187 *rol-6(e187) II*, *dpy-17(e164) III*, YA1022 *pot-1(tm1620) III*, *unc-32(e189) III*, and YA1026 *pot-3(ok1530) III*.

The *pot-1* mutation was outcrossed *vs.* an outcrossed stock of *dpy-17unc-32*. *pot-2* and *pot-1*::*mCherry* lines were outcrossed *vs.* outcrossed stocks of *unc-52* or *rol-6*, respectively. Freshly isolated homozygous F2 lines were established for analysis.

To create *pot-1*; *pot-2* double mutants, a *pot-1*; *unc-52* double mutant and a *pot-2*; *dpy-17unc-32* triple mutant were first created; phenotypically wild-type F2 progeny of *unc-52 / pot-2*; *pot-1 / dpy-17*, *unc-32* F1 heterozygotes were selected; and the strains that segregated only phenotypically wild-type F3 progeny were retained for analysis.

To create the *trt-1*; *pot-1* and *trt-1*; *pot-2* double mutants, *dpy-5unc-55*; *pot-1*, *dpy-5unc-55*; *pot-2*, *trt-1*; *dpy-17unc-32*, and *trt-1*; *unc-52* mutants were generated. Phenotypically wild-type F2 progeny of *dpy-5unc-55* / *trt-1*; *pot-1* / *dpy-17unc-32* or *dpy-5unc-55* / *trt-1*; *pot-2* / *unc-52* F1 heterozygotes were selected, and strains that segregated only phenotypically wild-type F3 progeny were retained for analysis.

To create *trt-1*; *pot-2*; *pot-1* triple mutants, *trt-1*; *pot-2*; *dpy-17unc-32* and *trt-1*; *unc-52*; *pot-1* triple mutants were first created, phenotypically wild-type F2 progeny were selected from *trt-1*; *pot-2 / unc-52*; *pot-1 / dpy-17unc-32* heterozygotes, and the strains that segregated only phenotypically wild-type F3 progeny were retained for analysis.

To place the *ypIn2 pot-1*::*mCherry* transgene into a *pot-1* mutant background, *ypIn2*; *dpy-17unc-32* hermaphrodites were crossed to *rol-6 /* +; *pot-1 / dpy-17unc-32* males, and phenotypically wild-type F2 progeny were singled from *rol-6 / ypIn2*; *pot-1 / dpy-17unc-32* F1, and F2 that segregated only phenotypically wild-type F3 progeny were retained for analysis. *ypIn2 pot-1*::*mCherry* was placed into the *pot-2* mutant background analogously, where *ypIn2 pot-1*::*mCherry unc-52* hermaphrodites were crossed to *rol-6 / +*; *pot-2 / unc-52* males and phenotypically wildtype F2 progeny were selected.

To create a *pot-1*::*mCherry* strain that expressed *GFP*::*Histone H2B* from the transgene insertion *ruIs32*, N2 males were crossed to hermaphrodites of the strain TH32 *unc-119ed3*; *ddIs6[tbg-1*::*GFP + unc-119(+)]*; *ruIs32[unc-119(+) pie-1*::*GFP*::*H2B]* (Desai *et al.* 2003), and the resultant F1 males were crossed with *pot-1*::*mCherry* hermaphrodites. F1 cross-progeny were singled and allowed to self-fertilize, F2 progeny were singled from animals heterozygous for both *ruIs32[unc-119(+) pie-1*::*GFP*::*H2B]* and *pot-1*::*mCherry*, and F3 progeny homozygous for both transgenes were selected.

### Terminal restriction fragment length analysis

*C. elegans* genomic DNA was isolated using Gentra Puregene reagents (QIAGEN), digested with *Hin*fI enzyme (NEB), and separated on a 0.6% agarose gel at 1.5 V/cm. Southern blotting was performed using the DIG Wash and Block Buffer Set (Roche) following the manufacturer’s instructions. A telomere probe, corresponding to the *C. elegans* telomeric repeat TTAGGC, was synthesized and labeled with digoxigenin (DIG)-dUTPs using the PCR DIG Probe Synthesis Kit (Roche) following the manufacturer’s instructions.

### Telomere erosion rate calculation

The sizes of individual telomere bands, between 2 and 6 kb, that could be clearly followed for consecutive generations (and were distinct from bands corresponding to neighboring telomeres or to interstitial telomeric tracts) were calculated using semi-log graphs of molecular marker size and distance traveled from the well (Ahmed and Hodgkin 2000; Boerckel *et al.* 2007; Lowden *et al.* 2008; Meier *et al.* 2006, 2009). Data are presented as the mean ± SD.

### Transgene construction

All transgene constructs were made using the *MosI*-mediated single-copy insertion system that allows for the incorporation of a single copy of a transgene into one specific locus in the *C. elegans* genome (Frokjaer-Jensen *et al.* 2008). The *pot-1*::*mCherry* transgene was constructed using the Invitrogen Gateway Cloning kit using the positive selection marker *Cb-unc-119(+)*, a germline-specific promoter *daz-1*, full-length genomic *pot-1* sequence lacking a stop codon, *mCherry* sequence, and the *tbb-2* 3′ UTR. An extrachromosomal array consisting of this construct, *Pglh-2*::*Mos1 transposase*, and three fluorescent *mCherry* negative selection markers was introduced into *Mos-1(ttTi5605)*; *unc-119* worms via microinjection into the germline of young adults. Progeny of injected animals were screened for loss of the Unc phenotype and for the presence of mCherry fluorescence, suggesting successful transformation of the injected extrachromosomal array. Lines with successful transformants were further propagated and progeny were screened for loss of coinjection *mCherry* fluorescence markers but continued rescue of the Unc phenotype, indicating successful integration of the construct. Genomic DNA prepared from these lines was tested by PCR and DNA sequencing to confirm the presence of a single-copy insertion in the *Mos-1(ttTi5605)* insertion site on chromosome *II*. The *unc-119* mutation was removed from transgenic strains prior to analysis by crossing with *rol-6 / +* males, singling non-Rol, non-Unc F2 from F1 with Rol F2, choosing F2 that lacked *unc-119* homozygotes, and selecting against *rol-6*. Single-copy transgene insertions were designated *ypIn2 (Pdaz-1*::*pot-1*::*mCherry*::*tbb-2utr)* and *ypIn3 (Pdaz-1*::*pot-1*::*mCherry*::*tbb-2utr)*, which we refer to below as *pot-1*::*mCherry.B* and *pot-1*::*mCherry.C*, respectively.

### DAPI staining

One-day-old adult worms were soaked in 150 µL of a 400 ng/mL DAPI in ethanol solution for 30 min or until evaporated, rehydrated in 2 mL of M9 solution overnight at 4°, and mounted in 5 µL of fresh NPG/glycerol medium. Chromosome counts were performed under ×100 magnification and a 359 excitation wavelength using a Nikon Eclipse E800 microscope.

### POT-1::mCherry foci quantification

Live 1-day-old adult worms were mounted onto 2% agarose pads in 5 µL of tetramisole and Z stacks were taken within 2 hr of mounting under ×100 magnification and a 595-nm excitation wavelength using a Nikon Eclipse E800 microscope. Foci from individual nuclei were quantified by manually scanning through compiled Z stacks.

### C-circle quantification

The C-circle amplification assay was performed as previously described (Henson *et al.* 2009) with the following modifications: (1) the 96-nucleotide oligomer control was generated with a *C. elegans* telomeric sequence (5′ CCCATATCACTAA(GCCTAA)_12_CCTCAATTCCC 3′); (2) the DNA was resolved on an agarose gel and normalized by ethidium bromide staining, and amplified DNA was dot blotted onto a neutral nylon membrane (GE Healthcare Life Sciences) and probed with a telomeric G strand (TTAGGC)_3_ oligo conjugated to DIG at 37°; and (3) the membrane was washed as described for the DIG Wash and Block Buffer Set (Roche) at 37° and developed with ECF reagent (GE Life Sciences). Fluorescence signals were collected with a Typhoon Trio scanner (GE Life Sciences) and quantified with ImageQuant TL software (GE Life Sciences) using edge subtraction.

## Results

### POT-1 and POT-2 are negative regulators of telomere extension *in vivo*

We obtained strains harboring the deletions *pot-1(tm1620)* or *pot-2(tm1400)* from Shohei Mitani and verified the presence of homozygous deletions in these strains using the polymerase chain reaction. Southern blotting revealed long telomeres for genomic DNA isolated from *pot-1* or *pot-2* mutant strains that were propagated for varying numbers of generations (Figure 1B). In contrast, the *pot-3(ok1530)* deletion did not have an overt effect on telomere length (Figure 1B). Outcrossing of *pot-1* or *pot-2* mutations for 15 generations as heterozygotes, followed by isolation of homozygous mutant *pot-1* or *pot-2* strains, revealed normal telomere lengths in early generations followed by progressive telomere elongation (Figure 1C). Therefore, the telomere elongation phenotypes caused by *pot-1* and *pot-2* mutations are recessive and can be eliminated if the mutations are maintained as heterozygotes.

Telomeres from *pot-1* and *pot-2* mutant strains had qualitatively similar dynamics, suggesting that POT-1 and POT-2 may perform similar functions at telomeres. The rapid appearance of smeary, long telomeres, assessed from numerous, outcrossed lines of *pot-1* and *pot-2* single mutants or *pot-2*; *pot-1* mutants, precluded measurement of telomere elongation rates with errors of <100 bp/generation, although telomere elongation was qualitatively similar among the three genotypes (Figure 1, C and D; supporting information, Figure S1).

To confirm that the *pot-1(tm1620)* mutation was responsible for the telomere elongation phenotype of outcrossed *pot-1* strains, single-copy transgenes designed to express wild-type POT-1 fused to a fluorescent mCherry protein at its C terminus were created, outcrossed nine times, and crossed into a *pot-1(tm1620)* background that had been outcrossed 30 times. In contrast to *pot-1(tm1620)* mutants (Figure 1C), telomere lengths in independent *pot-1*::*mCherry*; *pot-1(tm1620)* strains remained constant over many generations (Figure 1E), indicating that the progressive telomere elongation phenotype of *pot-1(tm1620)* mutants is caused by the *pot-1* deletion rather than a tightly linked mutation. Moreover, telomeres did not progressively lengthen or shorten for independently outcrossed *pot-1*::*mCherry* strains in a wildtype *pot-1* background, indicating that the POT-1::mCherry fusion protein does not perturb the ability of endogenous POT-1 to regulate telomere length (Figure 1F). However, bulk telomere length was slightly longer than wildtype for strains containing a *pot-1*::*mCherry* transgene (Figure 1, D and F).

### POT-1 foci at *C. elegans* telomeres *in vivo*

**Figure 2:**
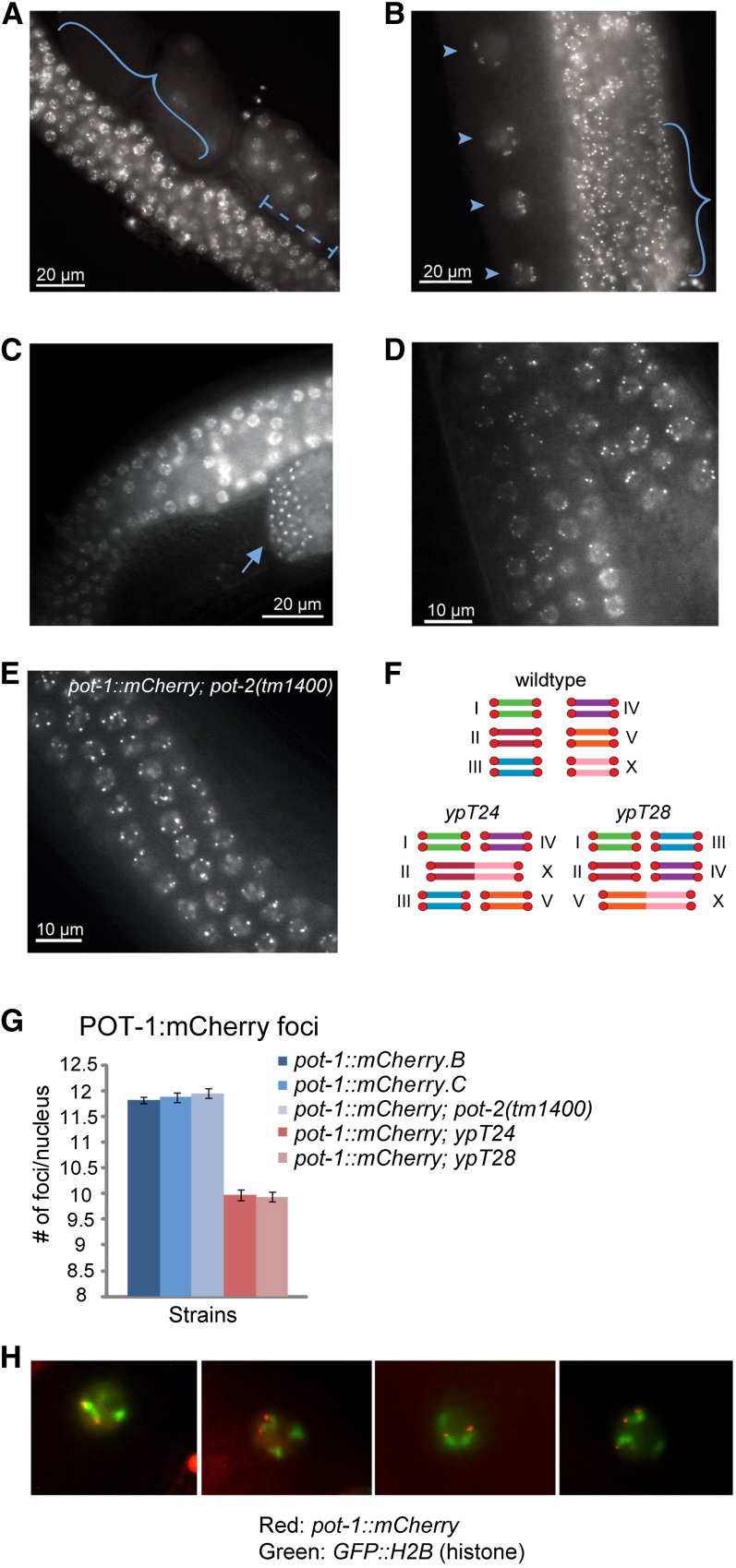
POT-1:mCherry localizes to telomeres as punctate foci independent of POT-2. Live imaging of *pot-1*::*mCherry* strains revealed germline-specific expression, including meiotic nuclei (A, B; solid brackets), mitotic nuclei (A; dashed brackets), oocytes (B; arrowheads), and sperm (C; arrow). (D) Representative image of *pot-1*::*mCherry*. (E) Representative image of *pot-1*::*mCherry*; *pot-2(tm1400)*. (F) A representation of six *C. elegans* chromosomes in wild-type and in two strains harboring end-to-end chromosomal fusions (*ypT24* and *ypT28*). Red circles at chromosome termini represent telomeres. (G) POT-1:mCherry foci were quantified in the meiotic nuclei of two independent wild-type strains, *pot-1*::*mCherry.B* (n = 83) and *C* (n = 54), in a *pot-2(tm1400)* mutant strain (n = 56), and in the strains *ypT24* (n = 30) and *ypT28* (n = 51). The close paring of both sister chromatids and homologous chromosomes in *C. elegans* oocytes precludes the resolution of telomeres from distinct homologous chromosomes and instead reveals 12 or 10 telomeric spots in wildtype or fusion strains, instead of 24 or 20, respectively. (H) Representative images of live *pot-1*::*mCherry*; *GFP*::*histone* animals demonstrate POT-1::mCherry localization at chromosome ends.

Live imaging of animals possessing *pot-1*::*mCherry* transgenes revealed strong punctate POT-1::mCherry foci within the nuclei of sperm, some oocytes, and at the nuclear periphery throughout the rest of the germline (Figure 2, A−C). POT-1::mCherry foci could be robustly quantified in meiotic pachytene nuclei near the bend of germline arms, where the six homologous chromosomes of *C. elegans* are synapsed and in late stages of meiotic recombination (Dernburg *et al.* 1998). Analysis of independent *pot-1*::*mCherry* transgene insertions, *pot-1*::*mCherry.B* and *pot-1*::*mCherry*.*C*, revealed approximately 12 foci per pachytene nucleus (11.8 ± 0.1; 11.9 ± 0.1; Figure 2G), which could plausibly correspond to chromosome termini of the six paired homologous chromosomes (Figure 2, D and F). Slightly fewer than the expected mean number of telomeric foci were observed, likely due to telomeres that were occasionally near one another within a nucleus, precluding them from being distinguished as distinct foci.

Immunoprecipitation experiments have previously shown that a POT-1::HA fusion protein can interact with telomeric DNA in *C. elegans* (Raices *et al.* 2008). To confirm that POT-1::mCherry foci occurred at telomeres, we used the well-characterized end-to-end chromosome fusions *ypT24* and *ypT28*, which were isolated from *C. elegans* strains that were deficient for telomerase and then crossed onto telomerase-positive genetic backgrounds. These chromosome fusions were created from two chromosome ends that had lost all (TTAGGC)_n_ telomeric repeat sequences as well as several thousand base pairs of subtelomeric DNA prior to being joined together (Lowden *et al.* 2008, 2011). Strains homozygous for *ypT24* and *ypT28 X*-autosome chromosome fusions harbor five homologous chromosomes and 10 chromosome termini (Figure 2F), and these chromosome fusions can be stably maintained in *C. elegans* due to the presence of holocentric chromosomes. *ypT24* and *ypT28* chromosome fusions were crossed with *pot-1*::*mCherry* to create *pot-1*::*mCherry*; *ypT24* and *pot-1*::*mCherry*; *ypT28* strains, and quantification of POT-1 fluorescent foci in these strains revealed approximately 10 meiotic foci per nucleus (10 ± 0.1; 9.9 ± 0.1; Figure 2G), indicating that POT-1:mCherry foci correspond to discrete chromosome termini that are not clustered in meiotic pachytene nuclei. In addition, POT-1::mCherry foci were observed at termini of some condensed chromosomes in diakinesis-stage oocyte nuclei, where chromosomes were marked by histone H2B::GFP expression (Figure 2H). However, oocyte nuclei displayed reduced numbers of POT-1::mCherry foci in comparison with pachytene nuclei, and high-resolution images of POT-1::mCherry foci at both ends of a bivalent were not observed in oocytes. POT-1::mCherry expression became attenuated and more diffuse in the oocyte closest to the spermatheca.

Our results demonstrate that POT-1 localizes to telomeres as small, quantifiable, nuclear domains and is unlikely to form foci at any other segment of the *C. elegans* genome. To our knowledge, this is the first demonstration that stable genome rearrangements can be employed to show that a telomere binding protein specifically interacts with telomeres *in vivo*. Telomere clustering has been reported as chromosomes pair during meiosis (Bass *et al.* 1997; Cooper *et al.* 1998; Scherthan *et al.* 1996; Yamamoto and Hiraoka 2001), a process that occurs in transition zone nuclei of the *C. elegans* germline. POT-1::mCherry foci became diffuse and were rarely discrete at this stage of germ cell development (Figure S2), possibly due to rapid chromosome movements as chromosomes pair.

In contrast to previous results suggesting different functions for POT-1 and POT-2 (Raices *et al.* 2008), the lack of a qualitative additive telomere elongation phenotype for *pot-1*; *pot-2* double mutants suggested a common function for their gene products (Figure 1D; Figure S1). Therefore, we assessed whether the telomeric localization of POT-1 was affected by POT-2 by quantifying POT-1::mCherry foci in live *pot-1*::*mCherry*; *pot-2(tm1400)* animals. Approximately 12 foci per meiotic pachytene nucleus were observed when *pot-2* was mutant (12 ± 0.1), and the POT-1::mCherry localization pattern was qualitatively similar throughout the germline in wild-type and *pot-2* mutant backgrounds (Figure 2, E and G). Thus, POT-2 did not have an obvious effect on the telomeric localization of POT-1.

The distal portion of the *C. elegans* germline is composed of a population of proliferating mitotic cells (Cinquin *et al.* 2010). In contrast to cells arrested in meiotic pachytene, quantification of POT-1::mCherry foci in mitotic nuclei revealed an average of 18.9 foci per nucleus (± 2 SD). We observed a broader range of foci per nucleus in the mitotic region (16−23, n = 31), in part due to the smaller size and denser clustering of the nuclei, which precluded the more precise resolution of POT-1::mCherry foci that was possible in large pachytene nuclei. As some mitotic nuclei displayed less than 24 POT-1::mCherry spots, weak telomere clustering is likely to occur in mitotic cells of *C. elegans*. However, the presence of 20−23 spots in some nuclei suggested that mitotic telomere clustering could either be transient or could vary with the cell cycle. Our *pot-1*::*mCherry* transgene was only expressed in germ cells, because it was driven by the germ cell−specific *pgl-3* promoter. Future analysis of telomere behavior in somatic cell types may be an interesting line of investigation.

### POT-1 and POT-2 repress telomerase activity at telomeres

**Figure 3:**
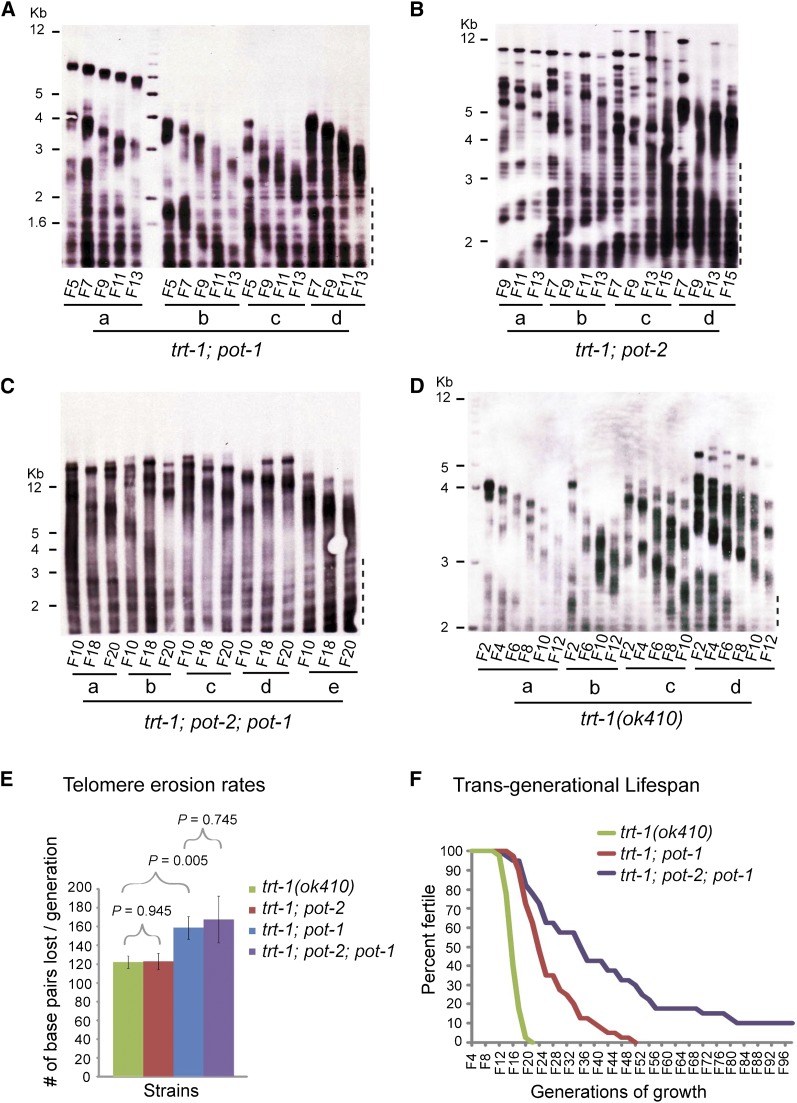
POT-1 and POT-2 negatively regulate telomerase-mediated telomere repeat addition. DNA collected from consecutive generations of (A) *trt-1*; *pot-1*, (B) *trt-1*; *pot-2*, (C) *trt-1*; *pot-1*; *pot-2*, and (D) *trt-1* mutant strains was submitted to terminal restriction fragment length analysis. (E) To measure shortening rates, telomeres that were 2−6 kb in size and could be accurately scored for changes in length were examined. Error bars represent the SEM, and *P* values were determined by the Student’s *t*-test. (F) Six animals per strain (n = 40) of the indicated genotypes were passaged weekly until sterility, where one week indicates two generations of growth. Dashed line to the right of the blots indicates internal telomeric sequences.

To ascertain whether the telomere elongation phenotype of *pot-1* and *pot-2* mutants is mediated by telomerase, we crossed the *pot-1* and *pot-2* mutations into a telomerase-deficient background by constructing double and triple mutants with a null allele of the telomerase reverse transcriptase, *trt-1(ok410)*. Telomeres shortened progressively in the absence of telomerase and *pot-1*, *pot-2* or both *pot-1* and *pot-2* (Figure 3, A−C). Therefore, telomere elongation of *pot-1* or *pot-2* single mutants depends on telomerase activity.

In the absence of telomerase, canonical DNA polymerases cannot maintain telomere length, resulting in a loss of telomere sequence with each cell division. Previous analysis of strains that are deficient for independent alleles of *trt-1*, for mutations in three additional *C. elegans* genes that are required for telomerase-mediated telomere maintenance, or for double mutants corresponding to *trt-1* and the former genes, has revealed consistent rates of telomere erosion of ~120 bp per generation for every genotype (Ahmed and Hodgkin 2000; Boerckel *et al.* 2007; Lowden *et al.* 2008; Meier *et al.* 2006, 2009). We asked whether POT-1 or POT-2 affected the rate of telomere erosion in the absence of telomerase by quantifying telomere shortening for *trt-1*; *pot-1* and *trt-1*; *pot-2* double mutants, for *trt-1*; *pot-2*; *pot-1* triple mutants and for *trt-1* single mutant controls. An enhanced telomere erosion rate was observed for *trt-1*; *pot-1* mutants (159 ± 12 bp/generation) in comparison to *trt-1* single mutants (122 ± 6 bp/generation; Figure 3D) or *trt-1*; *pot-2* double mutants (123 ± 9 bp/generation; Figure 3E). Therefore, POT-1, but not POT-2, protects telomeres from exacerbated erosion in the absence of telomerase. Moreover, *trt-1*; *pot-2*; *pot-1* telomeres shortened at a similar rate (168 ± 9 bp/generation) to *trt-1*; *pot-1* telomeres, indicating that POT-2 does not have an obvious telomere protection function in the absence of POT-1 (Figure 3E).

To study the effects of the modestly exacerbated telomere erosion observed in *pot-1* mutant strains that are deficient for telomerase, the onset of sterility (senescence) was quantified for *trt-1*; *pot-1* double mutants and *trt-1*; *pot-2*; *pot-1* triple mutants (n = 40 independent lines per strain). Both *trt-1*; *pot-1* double mutants (26.8+/−1.3 generations) and *trt-1*; *pot-2*; *pot-1* triple mutants (≥44 ± 3.6 generations) exhibited longer *trans*-generational lifespans (the number of generations until sterility) in comparison to *trt-1* mutant controls (16.7 ± 0.4 generations; *P* < 0.001; Student’s *t*-test), despite their faster rates of telomere erosion (Figure 3F). In addition, *trt-1*; *pot-2*; *pot-1* triple mutants exhibited a significantly longer average *trans*-generational lifespan than the *trt-1*; *pot-1* double mutants (*P* < 0.001; Student’s *t*-test), and some did not senesce (Figure 3F). Thus, although POT-1 mildly represses telomere shortening in the absence of telomerase, deficiency for *pot-1* or both *pot-1* and *pot-2* failed to enhance the onset of senescence in the absence of telomerase. This increased *trans*-generational lifespan is consistent with recent reports that either POT-1 or POT-2 can suppress the telomerase-independent telomere maintenance mechanism termed ALT (Cheng *et al.* 2012; Lackner *et al.* 2012).

**Figure 4:**
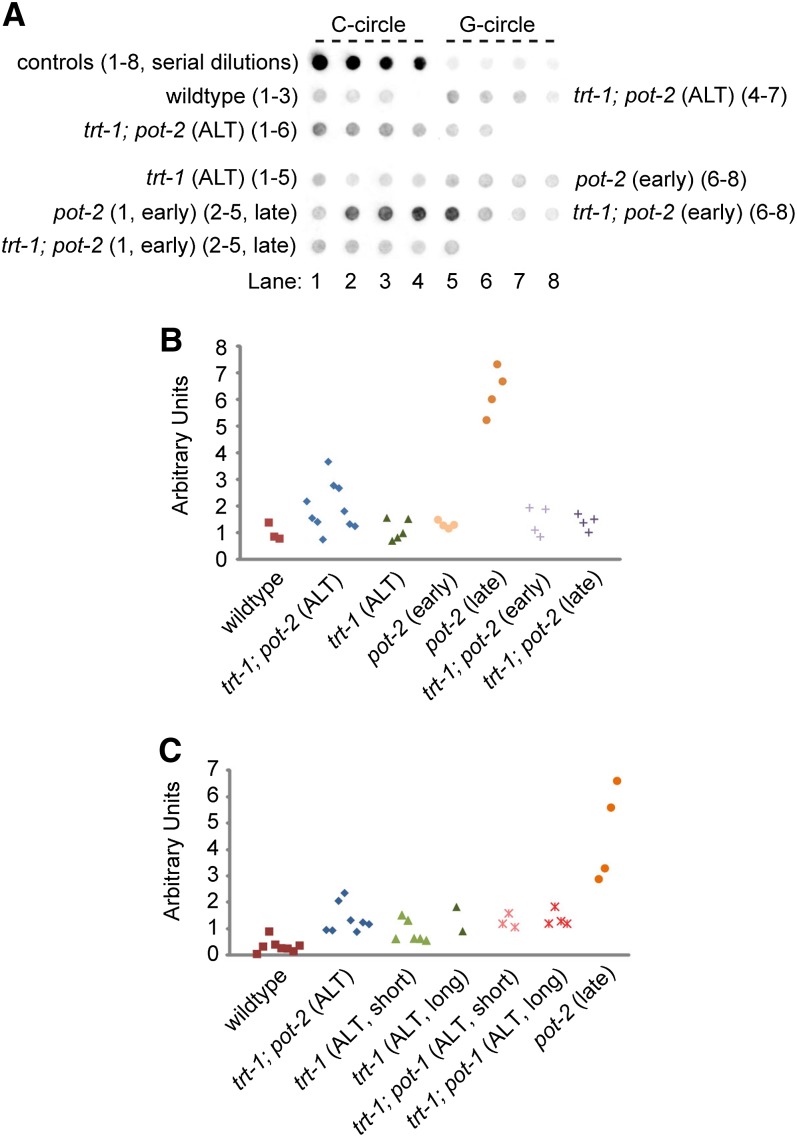
Telomeric C-circle levels increase in late-generation *pot-2* mutants. (A) A representative dot blot of a C-circle assay that was performed with DNA collected from multiple, independent mutant animals that were ALT, had grown for multiple generations (>20, “late”), or had grown for few generations (<6, “early”). (B) Quantification of C-circle assay amplification signals from (A). (C) Quantification of a C-circle assay with DNA from wild type, from late-generation *pot-2* single mutants with long telomerase, and from a variety of ALT strains that harbored short or long telomeres.

It has been previously reported that strains deficient for *pot-1* or *pot-2* with long telomeres display high levels of circular telomeric DNA (Raices *et al.* 2008). Further, mammalian cells that use the ALT telomere maintenance pathway possess high levels of telomeric C-circles, an established marker of ALT (Henson *et al*. 2009), and *C. elegans trt-1*; *pot-1* ALT strains display increased levels of telomeric C-circles in comparison with wild type (Lackner *et al.* 2012). We observed wild-type levels of telomeric C-circles in early-generation *pot-2* strains with short telomeres, and large 5- to 7-fold increases in C-circles in late-generation *pot-2* strains with long telomeres (Figure 4). Previously established *trt-1*; *pot-2* ALT strains with telomeres of normal lengths (Cheng *et al.* 2012) displayed little or no increases in C-circle formation, indicating that the high levels of C-circles in late-generation *pot-2* strains could require the presence of extremely long telomeres and possibly the activity of the telomerase reverse transcriptase. Consistent with the notion that telomerase could contribute to C-circle formation, *trt-1*; *pot-1* ALT strains with either short or very long telomeres displayed modestly elevated levels of C-circles (Figure 4C). Our data suggest that elevated C-circle levels could contribute to ALT, although the greatest levels of C-circles were observed for late-generation *pot-2* that possess long telomeres and were wild type for telomerase.

## Discussion

**Figure 5:**
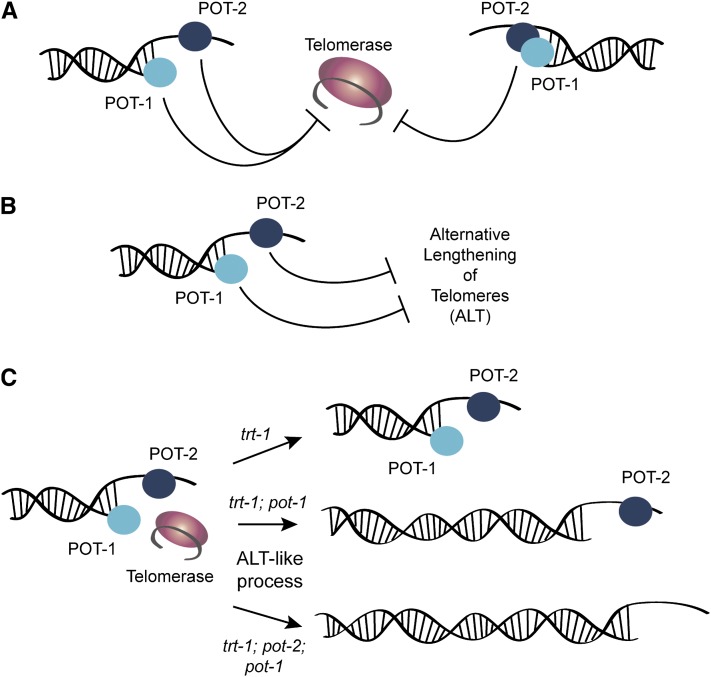
Model for interactions of POT-1 and POT-2 with telomerase and ALT. (A) POT-1 and POT-2 may repress telomerase-mediated telomere length maintenance via independent functions in the same process (left) or as a heterodimer (right). (B) In the absence of telomerase, POT-1 and POT-2 independently repress ALT-mediated telomere maintenance. (C) Early-generation telomerase mutants typically possess telomeres of normal lengths (top right), but deficiency for *trt-1* and *pot-1* (middle right) or for both *pot-1* and *pot-2* (bottom right) may initiate a rapid yet transient telomerase-independent telomere maintenance process that extends telomeres (length of double-stranded telomeric duplex is not drawn to scale), thereby allowing for an extended number of generations prior to senescence. A subset of *trt-1*; *pot-2*; *pot-1* triple mutants (bottom right) may activate an ALT-mediated telomere maintenance pathway, thereby allowing for immortalization in later generations.

POT1 is a multifunctional telomere capping protein, and the presence of four *C. elegans* genes with homology to human POT1 OB folds provides an opportunity to further elucidate the functions of POT1 in telomere biology. Here we show that *C. elegans* POT-1 and POT-2 single-stranded telomere-binding proteins negatively regulate telomerase-mediated telomere repeat addition. In addition, abrogation of telomerase activity in *pot-1* or *pot-2* mutants resulted in progressive telomere erosion. *In vitro* studies have previously shown that POT-1 preferentially interacts with single-stranded G-rich telomeric DNA, whereas POT-2 interacts with single-stranded C-rich telomeric DNA (Raices *et al.* 2008), suggesting that these proteins may play distinct roles at telomeres. Qualitatively similar telomere elongation dynamics for *pot-1* and *pot-2* mutants, and for *pot-1*; *pot-2* double mutants, suggest that these proteins may function in a similar manner to repress telomerase. Our data do not allow us to distinguish whether POT-1 and POT-2 function at distinct steps to repress telomerase or if this occurs via a POT-1/POT-2 heterodimer that possesses both OB1 and OB2 folds and could structurally resemble canonical POT1 proteins (Figure 5A).

We provide evidence for a distinct role for POT-1 in *C. elegans* telomere biology, as deficiency for *pot-1* but not *pot-2* modestly enhanced the rate of telomere erosion in *trt-1* mutants, suggesting a telomere capping function of POT-1. POT-1 is the sole *C. elegans* protein with an OB1 fold (Figure 1A) and can interact with non-terminal segments of single-stranded telomeric oligonucleotides *in vitro* (Raices *et al.* 2008). Thus, POT-1 may be well positioned to prevent resection of the 5′ end of the C-rich strand of the telomere. Consistent with our observations, mammalian POT1 has been shown to protect the 5′ end of the telomeric C-strand, which could be subjected to aberrant processing or resection in the absence of POT-1 (Hockemeyer *et al.* 2005). In contrast, the OB2 fold of POT-2 is predicted to interact with the 3′ end of single-stranded telomeric overhangs (Raices *et al.* 2008), which could be less relevant to protection or processing of telomeres in the absence of telomerase.

Deficiency for *pot-1* delayed the senescence phenotype of telomerase mutants, even though a modestly faster rate of telomere erosion occurred when *pot-1* was deficient. Recent independent reports have indicated that deficiency for *pot-1* or *pot-2* can promote the telomerase-independent telomere maintenance pathway ALT in *trt-1* mutants (Cheng *et al.* 2012; Lackner *et al.* 2012). POT-1 has been previously shown to repress C-circle formation (Raices *et al.* 2008), a bona fide marker of ALT, and here we show the same function for POT-2 (Figure 4), suggesting that DNA replication intermediates relevant to ALT may occur in animals lacking either of these proteins. We previously observed an ALT phenotype that allows telomerase mutants to escape senescence indefinitely, but only when hundreds of animals were transferred weekly (Cheng *et al.* 2012). In the present study, we transferred only 6 animals once a week, which we expected might preclude the onset of a full-blown ALT phenotype. We observed temporary extension of *trans*-generational lifespan for *trt-1* strains deficient for *pot-1*. However, a subset of *trt-1*; *pot-2*; *pot-1* triple mutants strains survived indefinitely when 6 larvae were transferred (Figure 3F). This indefinite survival phenotype, in conjunction with a longer *trans*-generational lifespan of *trt-1*; *pot-2*; *pot-1* triple mutants in comparison to *trt-1*; *pot-1* double mutants (Figure 3F), is consistent with our previous observation that POT-1 and POT-2 have independent roles in repressing a telomerase-independent telomere replication pathway (Figure 5B; Cheng *et al.* 2012), which may become fully engaged to robustly drive ALT in small populations of animals when both *pot-1* and *pot-2* are deficient. We observed that early generation *trt-1*; *pot-1* or *trt-1*; *pot-2*; *pot-1* mutants had longer initial telomere lengths than *trt-1* single mutants (Figure 3, A, C, and D), so initial telomere length may be largely responsible for the extended *trans*-generational lifespans of the double or triple mutants. Given that well-outcrossed *pot-1* or *pot-2* mutations with short telomere lengths were employed to establish these strains, we speculate that creation of *trt-1* strains that are deficient for *pot-1* or for both *pot-1* and *pot-2* promotes a telomerase-independent ALT-like pathway that rapidly extends telomeres in early generations (Figure 5C), but then dissipates allowing late-onset senescence to occur in most strains (Figure 3F).

The modest and progressive effects of deficiency for *pot-1* or *pot-2* are at odds with rapid and severe telomere phenotypes that occur in the presence of telomerase for *S. pombe pot1* mutants (Baumann and Cech 2001), for *P. patens pot1* mutants (Shakirov *et al.* 2010), and for expression of C-terminally truncated Pot2 in *Arabidopsis* (Shakirov *et al.* 2005; Surovtseva *et al.* 2007). More modest effects have been observed when Pot1 was abrogated in mammalian cells (Hockemeyer *et al.* 2006; Veldman *et al.* 2004). The four *C. elegans* POT1 homologs may each possess one or more functions of ancestral POT1, allowing their roles in telomere biology to be studied in detail. Finally, our *pot-1*::*mCherry* transgene allows for chromosome termini to be observed in living worms and may provide a useful tool for future studies of telomere and chromosome biology in *C. elegans*.

## Supplementary Material

Supporting Information

## References

[bib1] Ahmed S. Hodgkin J., 2000 MRT-2 checkpoint protein is required for germline immortality and telomere replication in *C. elegans*. Nature 403: 159–164 10646593 10.1038/35003120

[bib2] Armbruster B. N. Linardic C. M. Veldman T. Bansal N. P. Downie D. L., 2004 Rescue of an hTERT mutant defective in telomere elongation by fusion with hPot1. Mol. Cell. Biol. 24: 3552–3561 15060173 10.1128/MCB.24.8.3552-3561.2004 PMC381596

[bib3] Bass H. W. Marshall W. F. Sedat J. W. Agard D. A. Cande W. Z., 1997 Telomeres cluster de novo before the initiation of synapsis: a three-dimensional spatial analysis of telomere positions before and during meiotic prophase. J. Cell Biol. 137: 5–18 9105032 10.1083/jcb.137.1.5 PMC2139864

[bib4] Baumann P. Cech T. R., 2001 Pot1, the putative telomere end-binding protein in fission yeast and humans. Science 292: 1171–1175 11349150 10.1126/science.1060036

[bib5] Boerckel J. Walker D. Ahmed S., 2007 The *Caenorhabditis elegans* Rad17 homolog HPR-17 is required for telomere replication. Genetics 176: 703–709 17339221 10.1534/genetics.106.070201 PMC1893056

[bib6] Cheng C. Shtessel L. Brady M. M. Ahmed S., 2012 *Caenorhabditis elegans* POT-2 telomere protein represses a mode of alternative lengthening of telomeres with normal telomere lengths. Proc. Natl. Acad. Sci. USA 109: 7805–7810 22547822 10.1073/pnas.1119191109 PMC3356646

[bib7] Cinquin O. Crittenden S. L. Morgan D. E. Kimble J., 2010 Progression from a stem cell-like state to early differentiation in the C. elegans germ line. Proc. Natl. Acad. Sci. USA 107: 2048–2053 20080700 10.1073/pnas.0912704107 PMC2836686

[bib8] Colgin L. M. Baran K. Baumann P. Cech T. R. Reddel R. R., 2003 Human POT1 facilitates telomere elongation by telomerase. Curr. Biol. 13: 942–946 12781132 10.1016/s0960-9822(03)00339-7

[bib9] Cooper J. P. Watanabe Y. Nurse P., 1998 Fission yeast Taz1 protein is required for meiotic telomere clustering and recombination. Nature 392: 828–831 9572143 10.1038/33947

[bib10] Dernburg A. F. McDonald K. Moulder G. Barstead R. Dresser M., 1998 Meiotic recombination in *C. elegans* initiates by a conserved mechanism and is dispensable for homologous chromosome synapsis. Cell 94: 387–398 9708740 10.1016/s0092-8674(00)81481-6

[bib11] Desai A. Rybina S. Muller-Reichert T. Shevchenko A. Hyman A., 2003 KNL-1 directs assembly of the microtubule-binding interface of the kinetochore in *C. elegans*. Genes Dev. 17: 2421–2435 14522947 10.1101/gad.1126303 PMC218079

[bib12] Diotti R. Loayza D., 2011 Shelterin complex and associated factors at human telomeres. Nucleus 2: 119–135 21738835 10.4161/nucl.2.2.15135 PMC3127094

[bib13] Frokjaer-Jensen C. Davis M. W. Hopkins C. E. Newman B. J. Thummel J. M., 2008 Single-copy insertion of transgenes in Caenorhabditis elegans. Nat. Genet. 40: 1375–1383 18953339 10.1038/ng.248 PMC2749959

[bib14] Greider C. W. Blackburn E. H., 1989 A telomeric sequence in the RNA of Tetrahymena telomerase required for telomere repeat synthesis. Nature 337: 331–337 2463488 10.1038/337331a0

[bib15] Harley C. B. Futcher A. B. Greider C. W., 1990 Telomeres shorten during ageing of human fibroblasts. Nature 345: 458–460 2342578 10.1038/345458a0

[bib16] He H. Multani A. S. Cosme-Blanco W. Tahara H. Ma J., 2006 POT1b protects telomeres from end-to-end chromosomal fusions and aberrant homologous recombination. EMBO J. 25: 5180–5190 17053789 10.1038/sj.emboj.7601294 PMC1630418

[bib17] He H. Wang Y. Guo X. Ramchandani S. Ma J., 2009 Pot1b deletion and telomerase haploinsufficiency in mice initiate an ATR-dependent DNA damage response and elicit phenotypes resembling dyskeratosis congenita. Mol. Cell. Biol. 29: 229–240 18936156 10.1128/MCB.01400-08 PMC2612488

[bib46] Henson J. D. Cao Y. Huschtscha L. I. Chang A. C. Au A. Y., 2009 DNA C-circles are specific and quantifiable markers of alternative-lengthening-of-telomeres activity. Nat. Biotechnol. 27: 1181–1185 19935656 10.1038/nbt.1587

[bib18] Hockemeyer D. Sfeir A. J. Shay J. W. Wright W. E. de Lange T., 2005 POT1 protects telomeres from a transient DNA damage response and determines how human chromosomes end. EMBO J. 24: 2667–2678 15973431 10.1038/sj.emboj.7600733 PMC1176460

[bib19] Hockemeyer D. Daniels J. P. Takai H. de Lange T., 2006 Recent expansion of the telomeric complex in rodents: Two distinct POT1 proteins protect mouse telomeres. Cell 126: 63–77 16839877 10.1016/j.cell.2006.04.044

[bib20] Hockemeyer D. Palm W. Wang R. C. Couto S. S. de Lange T., 2008 Engineered telomere degradation models dyskeratosis congenita. Genes Dev. 22: 1773–1785 18550783 10.1101/gad.1679208 PMC2492664

[bib21] Kelleher C. Kurth I. Lingner J., 2005 Human protection of telomeres 1 (POT1) is a negative regulator of telomerase activity in vitro. Mol. Cell. Biol. 25: 808–818 15632080 10.1128/MCB.25.2.808-818.2005 PMC543404

[bib22] Kendellen M. F. Barrientos K. S. Counter C. M., 2009 POT1 association with TRF2 regulates telomere length. Mol. Cell. Biol. 29: 5611–5619 19651898 10.1128/MCB.00286-09 PMC2756888

[bib23] Kim N. W. Piatyszek M. A. Prowse K. R. Harley C. B. West M. D., 1994 Specific association of human telomerase activity with immortal cells and cancer. Science 266: 2011–2015 7605428 10.1126/science.7605428

[bib24] Kim S. H. Kaminker P. Campisi J., 1999 TIN2, a new regulator of telomere length in human cells. Nat. Genet. 23: 405–412 10581025 10.1038/70508 PMC4940194

[bib25] Lackner D. H. Raices M. Maruyama H. Haggblom C. Karlseder J., 2012 Organismal propagation in the absence of a functional telomerase pathway in *Caenorhabditis elegans*. EMBO J. 31: 2024–2033 22425786 10.1038/emboj.2012.61 PMC3343340

[bib26] Liu D. Safari A. O’Connor M. S. Chan D. W. Laegeler A., 2004 PTOP interacts with POT1 and regulates its localization to telomeres. Nat. Cell Biol. 6: 673–680 15181449 10.1038/ncb1142

[bib27] Loayza D. De Lange T., 2003 POT1 as a terminal transducer of TRF1 telomere length control. Nature 423: 1013–1018 12768206 10.1038/nature01688

[bib28] Lowden M. R. Meier B. Lee T. W. Hall J. Ahmed S., 2008 End joining at *Caenorhabditis elegans* telomeres. Genetics 180: 741–754 18780750 10.1534/genetics.108.089920 PMC2567377

[bib29] Lowden M. R. Flibotte S. Moerman D. G. Ahmed S., 2011 DNA synthesis generates terminal duplications that seal end-to-end chromosome fusions. Science 332: 468–471 21512032 10.1126/science.1199022 PMC4154375

[bib30] Meier B. Clejan I. Liu Y. Lowden M. Gartner A., 2006 trt-1 is the *Caenorhabditis elegans* catalytic subunit of telomerase. PLoS Genet. 2: e18 16477310 10.1371/journal.pgen.0020018 PMC1361356

[bib31] Meier B. Barber L. J. Liu Y. Shtessel L. Boulton S. J., 2009 The MRT-1 nuclease is required for DNA crosslink repair and telomerase activity in vivo in *Caenorhabditis elegans*. EMBO J. 28: 3549–3563 19779462 10.1038/emboj.2009.278 PMC2782091

[bib32] Raices M. Verdun R. E. Compton S. A. Haggblom C. I. Griffith J. D., 2008 *C. elegans* telomeres contain G-strand and C-strand overhangs that are bound by distinct proteins. Cell 132: 745–757 18329362 10.1016/j.cell.2007.12.039

[bib33] Scherthan H. Weich S. Schwegler H. Heyting C. Harle M., 1996 Centromere and telomere movements during early meiotic prophase of mouse and man are associated with the onset of chromosome pairing. J. Cell Biol. 134: 1109–1125 8794855 10.1083/jcb.134.5.1109 PMC2120985

[bib34] Shakirov E. V. Surovtseva Y. V. Osbun N. Shippen D. E., 2005 The Arabidopsis Pot1 and Pot2 proteins function in telomere length homeostasis and chromosome end protection. Mol. Cell. Biol. 25: 7725–7733 16107718 10.1128/MCB.25.17.7725-7733.2005 PMC1190295

[bib35] Shakirov E. V. Perroud P. F. Nelson A. D. Cannell M. E. Quatrano R. S., 2010 Protection of Telomeres 1 is required for telomere integrity in the moss *Physcomitrella patens*. Plant Cell 22: 1838–1848 20515974 10.1105/tpc.110.075846 PMC2910979

[bib36] Sharma H. W. Sokoloski J. A. Perez J. R. Maltese J. Y. Sartorelli A. C., 1995 Differentiation of immortal cells inhibits telomerase activity. Proc. Natl. Acad. Sci. USA 92: 12343–12346 8618897 10.1073/pnas.92.26.12343 PMC40353

[bib37] Smogorzewska A. van Steensel B. Bianchi A. Oelmann S. Schaefer M. R., 2000 Control of human telomere length by TRF1 and TRF2. Mol. Cell. Biol. 20: 1659–1668 10669743 10.1128/mcb.20.5.1659-1668.2000 PMC85349

[bib38] Surovtseva Y. V. Shakirov E. V. Vespa L. Osbun N. Song X., 2007 Arabidopsis POT1 associates with the telomerase RNP and is required for telomere maintenance. EMBO J. 26: 3653–3661 17627276 10.1038/sj.emboj.7601792 PMC1949013

[bib39] van Steensel B. de Lange T., 1997 Control of telomere length by the human telomeric protein TRF1. Nature 385: 740–743 9034193 10.1038/385740a0

[bib40] Veldman T. Etheridge K. T. Counter C. M., 2004 Loss of hPot1 function leads to telomere instability and a cut-like phenotype. Curr. Biol. 14: 2264–2270 15620654 10.1016/j.cub.2004.12.031

[bib41] Wang F. Podell E. R. Zaug A. J. Yang Y. Baciu P., 2007 The POT1–TPP1 telomere complex is a telomerase processivity factor. Nature 445: 506–510 17237768 10.1038/nature05454

[bib42] Wu L. Multani A. S. He H. Cosme-Blanco W. Deng Y., 2006 Pot1 deficiency initiates DNA damage checkpoint activation and aberrant homologous recombination at telomeres. Cell 126: 49–62 16839876 10.1016/j.cell.2006.05.037

[bib43] Yamamoto A. Hiraoka Y., 2001 How do meiotic chromosomes meet their homologous partners?: lessons from fission yeast. Bioessays 23: 526–533 11385632 10.1002/bies.1072

[bib44] Ye J. Z. de Lange T., 2004 TIN2 is a tankyrase 1 PARP modulator in the TRF1 telomere length control complex. Nat. Genet. 36: 618–623 15133513 10.1038/ng1360

[bib45] Ye J. Z. Hockemeyer D. Krutchinsky A. N. Loayza D. Hooper S. M., 2004 POT1-interacting protein PIP1: a telomere length regulator that recruits POT1 to the TIN2/TRF1 complex. Genes Dev. 18: 1649–1654 15231715 10.1101/gad.1215404 PMC478187

