# Supplementary material for: Caenorhabditis elegans POT-1 and POT-2 Repress Telomere Maintenance Pathways
Source: G3 (Bethesda). 2013 Feb 1;3(2):305–13. doi: 10.1534/g3.112.004440 (PMC3564990; doi:10.1534/g3.112.004440)
Supplement: Supporting Information [file supp_3_2_305__index.html]

Supporting Information 

# *Caenorhabditis elegans* POT-1 and POT-2 Repress Telomere Maintenance Pathways

## Supporting Information for Shtessel *et al.*, 2013

**Files in this Data Supplement:**

- Supporting Information - Figures S1 and S2 (PDF, 816 KB)
- Figure S1 - Southern blotting of independent lines of *pot-1; pot-2* and *pot-2* mutants reveals qualitatively similar telomere elongation dynamics (PDF, 515 KB)
- Figure S2 - POT-1::mCherry localization in transition zone germline nuclei (PDF, 277 KB)
