# Supplementary material for: Caenorhabditis elegans POT-1 and POT-2 Repress Telomere Maintenance Pathways
Source: G3 (Bethesda). 2013 Feb 1;3(2):305–13. doi: 10.1534/g3.112.004440 (PMC3564990; doi:10.1534/g3.112.004440)
Supplement: Supporting Information [file supp_3.2.305_0044440SI.pdf]

G3 · Genes | Genomes | Genetics

C. elegans POT-1 and POT-2 repress telomere maintenance pathways

Ludmila Shtessel[*,§], Mia Rochelle Lowden[*,**], Chen Cheng[*,§§], Matt Simon[*,§], Kyle Wang[*] and Shawn Ahmed[*,§,**]

[*]Department of Genetics, [§]Curriculum in Genetics and Molecular Biology, [**]Department of Biology, University of North Carolina, Chapel Hill, NC 27599-3280, USA

[§§]Current Address: Department of Chemistry, University of California, Berkeley, CA 94720, USA

DOI: 10.1534/g3.112.004440

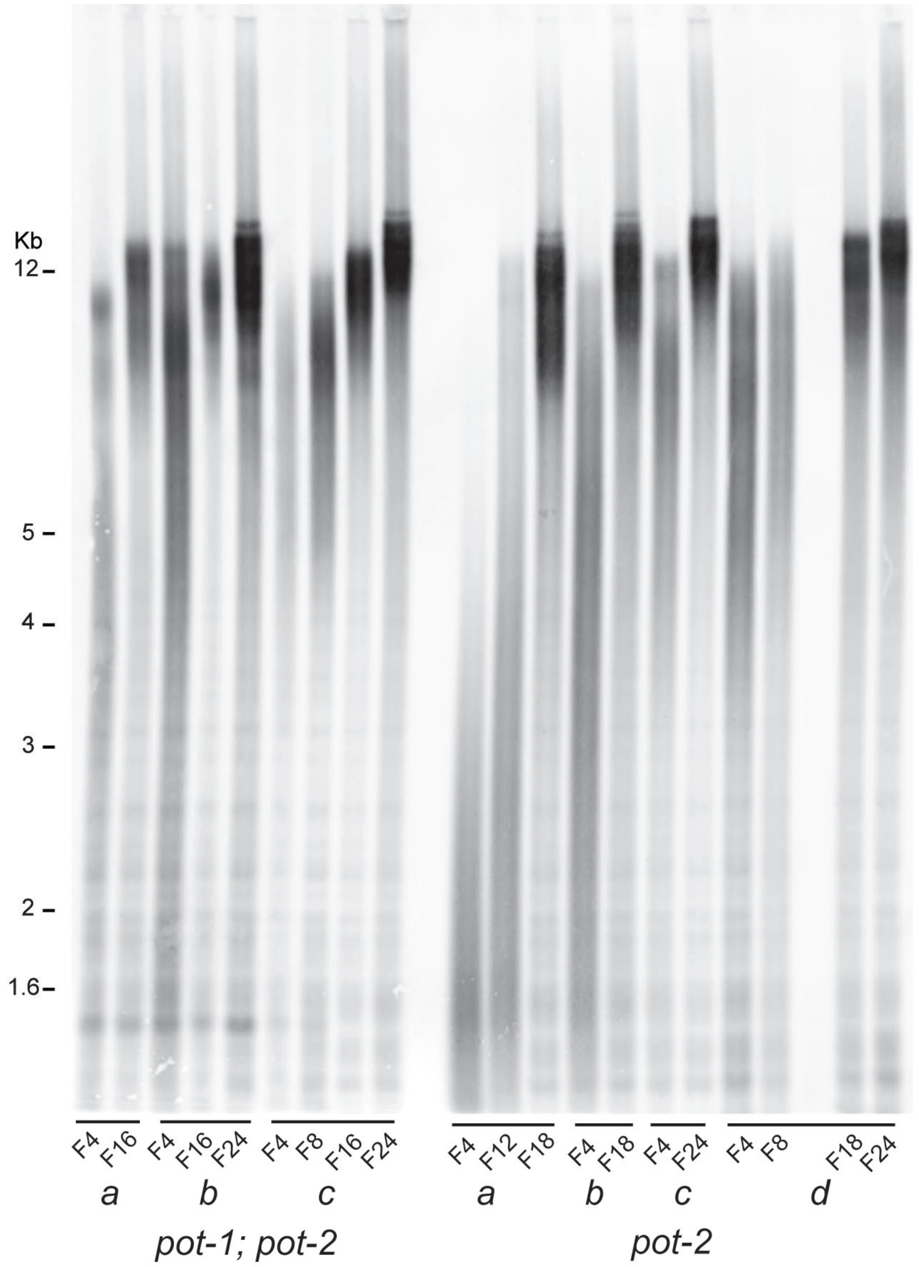

Figure S1. Southern blotting of independent lines of *pot-1; pot-2* and *pot-2* mutants reveals qualitatively similar telomere elongation dynamics.

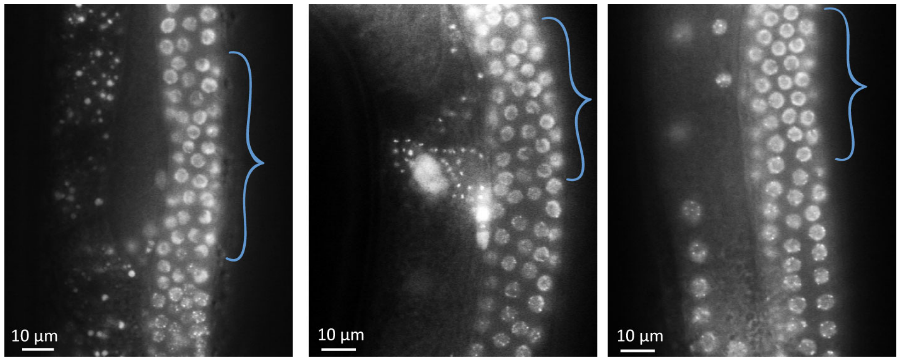

Figure S2. POT-1::mCherry localization in transition zone germline nuclei. POT-1::mCherry fluorescence is more diffuse in transition zone nuclei (brackets), where chromosomes begin to pair and enter meiosis.
