# Supplementary material for: Caenorhabditis elegans POT-1 and POT-2 Repress Telomere Maintenance Pathways
Source: G3 (Bethesda). 2013 Feb 1;3(2):305–13. doi: 10.1534/g3.112.004440 (PMC3564990; doi:10.1534/g3.112.004440)
Supplement: Supporting Information [file supp_3.2.305_FigureS1.pdf]

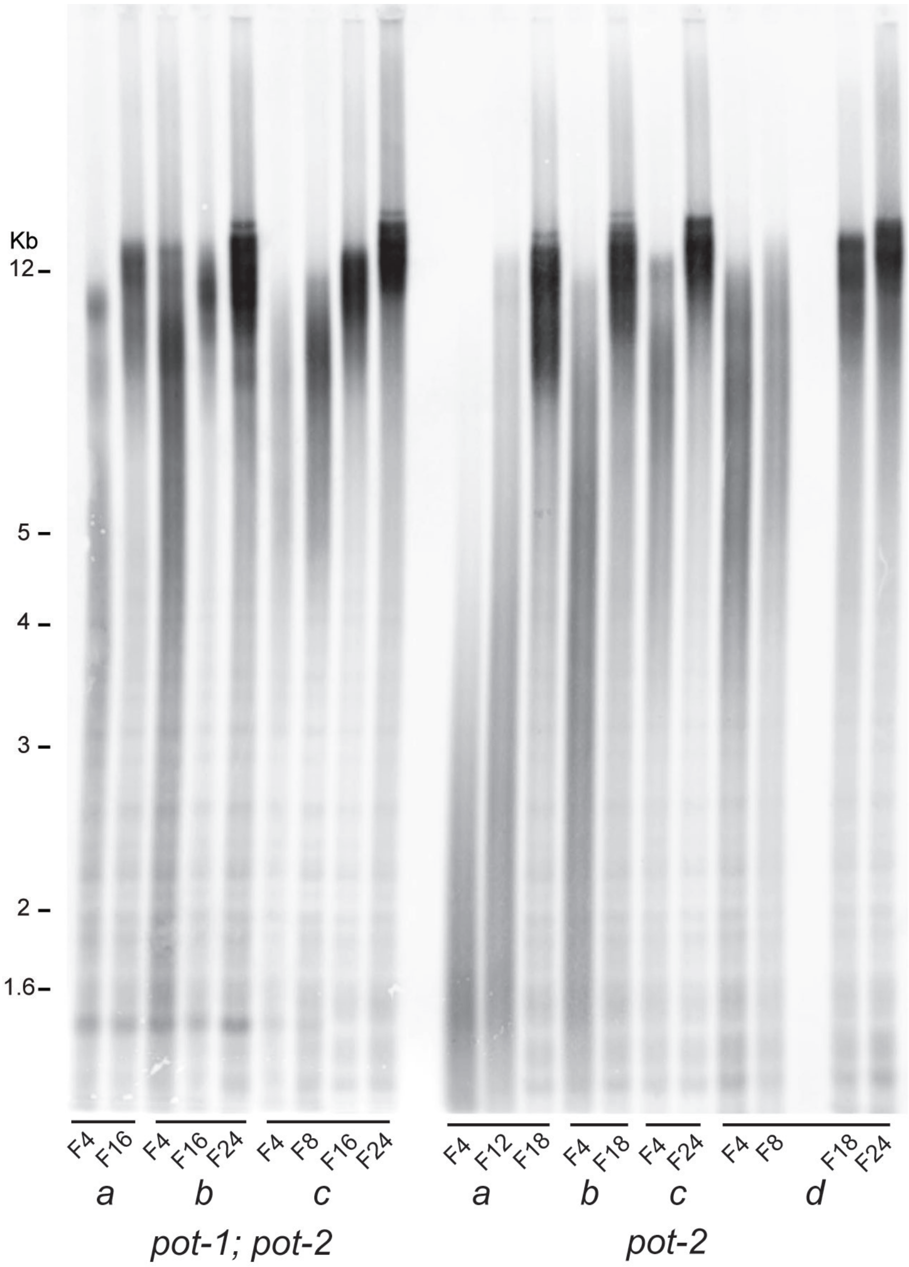

Figure S1. Southern blotting of independent lines of *pot-1; pot-2* and *pot-2* mutants reveals qualitatively similar telomere elongation dynamics.
