# Supplementary material for: Caenorhabditis elegans POT-1 and POT-2 Repress Telomere Maintenance Pathways
Source: G3 (Bethesda). 2013 Feb 1;3(2):305–13. doi: 10.1534/g3.112.004440 (PMC3564990; doi:10.1534/g3.112.004440)
Supplement: Supporting Information [file supp_3.2.305_FigureS2.pdf]

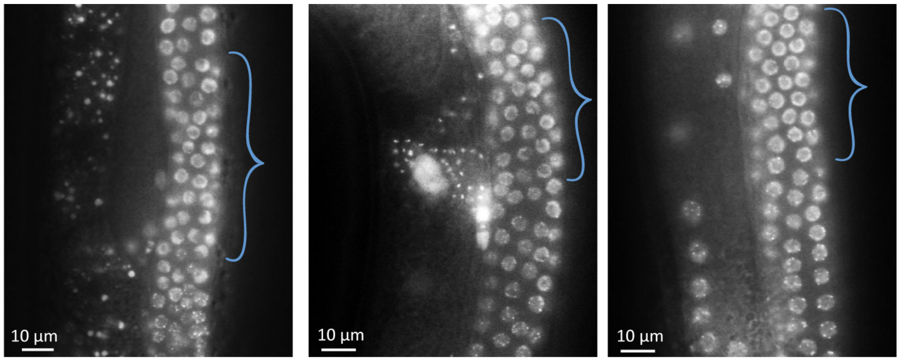

Figure S2. POT-1::mCherry localization in transition zone germline nuclei. POT-1::mCherry fluorescence is more diffuse in transition zone nuclei (brackets), where chromosomes begin to pair and enter meiosis.
